# Structural Basis for Activity Regulation and Substrate Preference of Clostridial Collagenases G, H, and T*[Fn FN2]

**DOI:** 10.1074/jbc.M112.448548

**Published:** 2013-05-23

**Authors:** Ulrich Eckhard, Esther Schönauer, Hans Brandstetter

**Affiliations:** From the Division of Structural Biology, Department of Molecular Biology, University of Salzburg, Billrothstrasse 11, A-5020 Salzburg, Austria

**Keywords:** Protease, Protein Degradation, Protein Structure, Proteolytic Enzymes, X-ray Crystallography, Collagenase, Metal Regulation

## Abstract

Clostridial collagenases are among the most efficient enzymes to degrade by far the most predominant protein in the biosphere. Here we present crystal structures of the peptidases of three clostridial collagenase isoforms (ColG, ColH, and ColT). The comparison of unliganded and liganded structures reveals a quaternary subdomain dynamics. In the unliganded ColH structure, this globular dynamics is modulated by an aspartate switch motion that binds to the catalytic zinc. We further identified a calcium binding site in proximity to the catalytic zinc. Both ions are required for full activity, explaining why calcium critically affects the enzymatic activity of clostridial collagenases. Our studies further reveal that loops close to the active site thus serve as characteristic substrate selectivity filter. These elements explain the distinct peptidolytic and collagenolytic activities of these enzymes and provide a rational framework to engineer collagenases with customized substrate specificity as well as for inhibitor design.

## Teaser

**Background:** Bacterial collagenases degrade collagen substrates with high efficiency yet varying specificity.

**Results:** The newly identified calcium site, aspartate switch, and conformational selectivity filter regulate substrate access to the active sites of these collagenases.

**Conclusion:** The unanticipated dynamics of the substrate recognition sites plus zinc occupancy combine to tune the enzymatic activity.

**Significance:** The crystal structures provide a rational framework to understand and optimize the isoform-dependent collagenase activities.

## Introduction

**FIGURE 1.**
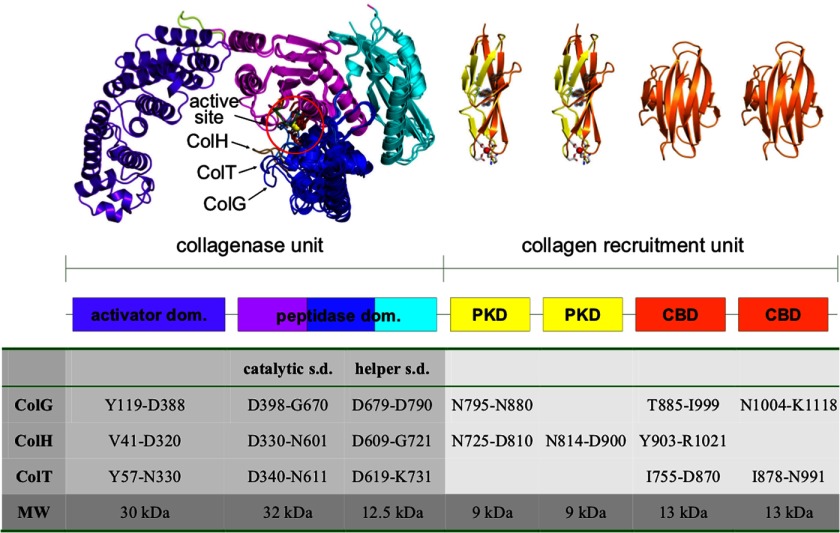
**Domain organization and quaternary architecture of mature clostridial collagenases.** Clostridial collagenases are composed of two functional units, the collagenase and the collagen recruitment unit. The saddle-shaped collagenase unit is composed of an activator and a peptidase domain. The latter is further segmented into an upper (*magenta*) and lower half-domain (*blue*), flanked by a helper subdomain (*cyan*). The collagen recruitment unit consists of up to two PKD-like domains (*yellow*) and up to three CBDs (*orange*). The catalytic zinc ion and the catalytic residues are shown in a *ball-and-stick representation* (*circled*). In the figure, the peptidase domains of ColG, ColH, and ColT are *superimposed*. (Sub)domain boundaries of collagenase G, H, and T are given in *tabular form* (one-letter amino acid codes). For the CBD model, Protein Data Bank entry 2O8O was used.

Clostridia employ collagenases for nutrition (*e.g.* saprophytic strains) and host colonization (1–3). They are large multidomain proteins of ∼115 kDa belonging to the gluzincin superfamily of metalloproteases (4). They all share a common HE*XX*H zinc binding motif complemented by an additional glutamate located 28–30 amino acids downstream, but the composition of their collagen recruitment domains varies. This can be exemplified by collagenase G (ColG)^4^ from *Clostridium histolyticum* and ColA from *Clostridium perfringens*, both of which possess one polycystic kidney disease (PKD)-like domain and two collagen binding domains (CBDs), whereas ColT from *Clostridium tetani* lacks the former, and ColH from *C. histolyticum* possesses two PKD-like domains but only one CBD (3, 5, 6) (Fig. 1). The recently determined crystal structure of ColG revealed a saddle-shaped architecture with a distinct segmentation of the N-terminal collagenase unit (Fig. 1). The collagenase unit is capable of degrading native collagen in the absence of the accessory domains within the C-terminally located collagen recruitment unit *in vitro* (7). It was further shown that the C-terminal saddle flap (Asp^398^–Asp^788^) harboring the active site could cleave the peptidic collagenase substrate *N*-[3-(2-furylacryloyl)]-l-leucyl-glycyl-l-prolyl-l-alanine (FALGPA) but could not degrade collagen. Thus, the peptidases studied here have no collagenolytic activity. The domain nomenclature reflects the functional assignment (Fig. 1).

Although vertebrate collagenases show a distinct substrate specificity and make only a single scission across all three collagen α-chains, bacterial collagenases hydrolyze native collagen at multiple sites and are capable of full degradation (3, 8–9). Based on the ratio of the activities toward synthetic peptides and native collagen, collagenases from *C. histolyticum* were divided into two distinct classes (10–12). Significantly, hydrolysis rates of ColG could be enhanced with substrates extending to the P4 residue, resulting in substrate cleavage rates by ColG comparable with those by ColH (12). The crystal structure analysis of ColG suggested that N-terminally extended substrates assist in their hydrolysis by employing additional interactions with the edge strand in ColG (7).

In addition to the active site zinc, clostridial collagenases were reported to require calcium for both their peptidolytic and collagenolytic activity (10, 13). Atomic absorption spectroscopy indicated 6.8 and 5.1 Ca^2+^ sites in ColG and ColH, respectively (10), but only the two calcium binding sites in the CBD were structurally elucidated (14). The lack of calcium binding in the ColG crystal structure can be explained by the presence of 0.1 m citrate in the crystallization buffer (7).

The interest in collagenases has been fostered by their multiple biotechnological and medical applications. For instance, the enzymes of *C. histolyticum* were recently approved as drugs to break down the tough cords in morbus Dupuytren (15–16) and are widely used in human cell islet isolation (17, 18) and wound debridement (19).

Here we present the crystal structures of the peptidase domains of ColH and ColT and the collagenase unit of ColG in the presence and absence of the peptidic inhibitor isoamylphosphonyl-Gly-Pro-Ala bound to the active site. The comparison of these homologues reveals differences in domain breathing motions and novel regulatory elements. Finally, we find that calcium and zinc are required for full peptidolytic activity, which can be explained by the spatial proximity of these metal binding sites in the peptidase domain.

## EXPERIMENTAL PROCEDURES

### 

#### 

##### Materials

All enzymes were purchased from Fermentas (St. Leon-Rot, Germany). Primers were obtained from Sigma-Aldrich; sequence analysis was performed at Eurofins MWG Operon (Ebersberg, Germany). All reagents were of the highest standard available from AppliChem (Darmstadt, Germany).

##### Cloning

Based on the crystal structure of the collagenase unit of ColG (Protein Data Bank entries 2Y3U, 2Y6I, and 2Y50) (7) and sequence alignments via ClustalW (20), we designed peptidase domain constructs of collagenase H from *Clostridium histolyticum* (Leu^331^–Gly^721^; numbering according to Swiss-Prot entry Q46085) and collagenase T from *C. tetani* (Asp^340^–Lys^731^; numbering according to Swiss-Prot entry Q899Y1). The peptidase domains were PCR-amplified from the respective full-length constructs (5) and cloned into a modified pET-15b vector, encoding for an N-terminal His_6_ tag, followed by a tobacco etch virus protease recognition site (ENLYFQ*X*GGT) and the enzyme-specific starting sequence.

Primers used were as follows: ColH-fwd, 5′-ACGTggtaccTTAGATAAGTTTAAAAAGGAAGG-3′; ColH-rev, 5′-ACGTggatccTTAACCTTCATTTGGTAAATATCC-3′; ColT-fwd, 5′-ACGTggtaccGACATTAATAAGTTAATAGAAGAAGG-3′; and ColT-rev, 5′-ACGTggatccTTATTTATTATGAGATAATAATC-3′. The collagenase unit of ColG was cloned as described previously (21). The PKD-like domain constructs of ColG Ile^792^–Asn^880^, Asn^795^–Asn^880^, and Ile^799^–Asn^880^ were cloned using the primers Ile^792^–Asn^880^-fwd (5′-ATCGggtaccATTAGTAACAATAAGGCTCC-3′), Asn^795^–Asn^880^-fwd (5′-ATGCggtaccAATAAGGCTCCAATAGCAAAGG-3′), Ile^799^–Asn^880^-fwd (5′-ATCGggtaccATAGCAAAGGTAACTGGACC-3), and the joint reverse primer 5′-AGCCggatccTTAGTTCTTT with the construct hG2 (5) as template. Restriction sites encoding for KpnI and BamHI are represented in lowercase letters. PCR products were purified with the MinElute PCR purification kit (Qiagen) and digested with the appropriate restriction enzymes, ligated overnight under standard conditions, and introduced into XL2-Blue (Stratagene) cells via electroporation. All constructs were confirmed by DNA sequencing prior to protein expression.

##### Expression and Purification

The peptidase domain constructs of ColH and ColT were expressed in soluble form in *Escherichia coli* BL21(DE3) cells at 37 °C in 2-liter baffled flasks in LB medium. The expression typically yielded 15 mg of pure protein from 600 ml of cell culture. All purification steps were carried out at 4 °C. The three-step purification included immobilized metal affinity chromatography, tag removal, rechromatography, and size exclusion chromatography as a final polishing step, as described in more detail elsewhere (5, 21). Protein expression and purification of the PKD-like domain constructs and of the collagenase unit were performed as described previously (5, 7, 22). Protein concentrations were assayed by detecting the absorbance at 280 nm. The molar extinction coefficients were calculated using the ProtParam tool available at ExPASy (23). The recombinant proteins migrated on a denaturing SDS-polyacrylamide gel with an apparent molecular mass of ∼79, 45, and 9 kDa for the collagenase unit of ColG, the peptidase domains of ColH and ColT, and the PKD-like domain constructs, respectively, and were found to be at least 95% homogeneous.

##### Enzymatic Assay

Enzymatic assays with FALGPA as substrate were performed as described by van Wart and Steinbrink (13) and detailed in the manufacturer's protocol (AppliChem) with the following modifications: (i) to raise the buffer capacity, Tricine concentration and pH were increased to 250 mm and 8.2, respectively; (ii) calcium was omitted from the reaction buffer; and (iii) protein was used at final concentrations of 10 μg/ml, respectively, in a 30-μl reaction volume.

The individual FALGPA solutions were made from a 4.0 mm FALGPA stock solution prepared in reaction buffer, and the concentrations were verified in solution via UV absorbance at 305 nm (ϵ_305_ = 24.70 mm^−1^ cm^−1^).

A 1.0 m solution of ZnCl_2_ was prepared in double-distilled H_2_O, diluted 1:100 (10 mm stock) and 1:1000 (1.0 mm stock), whereby the latter was used for serial dilutions. A 5.0 m CaCl_2_ and a 0.5 m EDTA (at pH 8.0) stock solution were prepared in double-distilled H_2_O and used for serial dilutions. All dilutions were done with reaction buffer. Protein samples used for enzymatic characterization were the same as for crystallization but at a lower protein concentration. Metal-depleted protein was prepared via incubation with 10 mm EDTA or 10 mm 1,10-phenanthroline overnight at 4 °C and rebuffered on a NAP-5 desalting column (GE Healthcare) pre-equilibrated with 250 mm Tricine, 400 mm NaCl, pH 8.2 (basic reaction buffer) before analysis. The protein stock solution of 0.1 mg/ml concentration was prepared in the reaction buffer (basic reaction buffer supplemented with the indicated CaCl_2_ and ZnCl_2_ concentrations) and incubated for 30 min at 4 °C prior to the measurements. All measurements were carried out at 25 °C, and the decrease in absorbance upon substrate cleavage was monitored at 345 nm in an Infinite M200 plate reader (Tecan).

All experiments were performed in triplicates and repeated at least twice. For *K*_*m*_ and *V*_max_ measurements, 14 different substrate concentrations were used ranging from 28 μm to 3.5 mm. Initial reaction velocities were calculated using nonlinear regression (24). The data were fitted to the Michaelis-Menten equation by nonlinear regression analysis using GraphPad Prism version 5.00 software (GraphPad Software).

##### Crystallization, Data Collection, and Structure Determination

Crystallization conditions were screened by the sitting drop vapor diffusion method using various commercial screens at 20 °C. Drops were prepared by mixing a 100-nl reservoir with a 200-nl protein solution containing, in the case of the peptidase domain constructs, 20 mg/ml protein in 10 mm Tris, pH 7.5, and 10 mm NaCl and, in case of the PKD-like domain constructs, 70 mg/ml protein in 10 mm Tris, pH 8.0, 25 mm NaCl, and 10 mm CaCl_2_. For pipetting, a Hydra II Plus One (Matrix Ltd.) liquid-handling system was used.

Crystals of the peptidase domain of ColH were obtained after 10 days in 20% PEG 3350 and 0.1 m sodium malonate, sodium formate, or sodium succinate, pH 7.0, and optimized by varying the initial crystallization conditions to 22.5% PEG 3350 and 0.15 m sodium formate at pH 7.25. Crystals of the ColT peptidase domain were obtained in 25% PEG 3350, 0.1 m HEPES, pH 7.5, and optimized to 25% PEG 3350, 0.1 m MES, pH 6.5, and 0.1 m NaCl. Crystals of the peptidase domains were harvested after cryoprotection by raising the PEG concentration to 30% in cryoloops, followed by flash cooling in a stream of nitrogen gas at 100 K.

Crystals of the PKD-like domain constructs Asn^795^–Asn^880^ and Ile^792^–Asn^880^ were harvested from 30% PEG 2000 monomethyl ether, 0.1 m potassium thiocyanate without any cryoprotection. For the collagenase unit and the PKD-like domain Ile^799^–Asn^880^, crystallization conditions and cryoprotection were as reported previously (7, 21, 22).

Crystals were screened in house with respect to diffraction limit and transported to the beamlines ID14-4 and ID29 at the European Synchrotron Radiation Facility (ESRF, Grenoble, France) for high resolution data collection. The oscillation angle was set to 0.5 and 0.25°, and the exposure time was set to 1.0 and 0.125 s/frame, respectively. A total of 360 and 720 images were recorded and processed using iMosflm (25) and Scala (26) within the CCP4 suite (27). Data collection and processing statistics are summarized in Table 1. A polyalanine chain trace of the peptidase domain of ColG (Asp^398^–Gly^790^; Protein Data Bank entry 2Y3U) was used for molecular replacement using the program Phaser (28). The model was iteratively improved by a combination of refinement using REFMAC5 (29) and manual building with COOT (30). The final stages of refinement were carried out by using bulk solvent correction, anisotropic scaling, and translation/libration/screw groups (31, 32) to model large scale thermal motions. The progress of refinement was cross-validated throughout using *R*_free_ with 5% of the unique reflections excluded from model refinement and map calculation. Refinement statistics are summarized in Table 1. Molecular figures were created with PyMOL (33). Contact areas were calculated by using the PISA server (34).

##### Structure Validation

The quality of all models was checked using the programs PROCHECK (35), WHATCHECK (36), and NQ-Flipper (37).

## RESULTS

### 

#### 

##### Sequence Comparisons of Functional Key Elements

**FIGURE 2.**
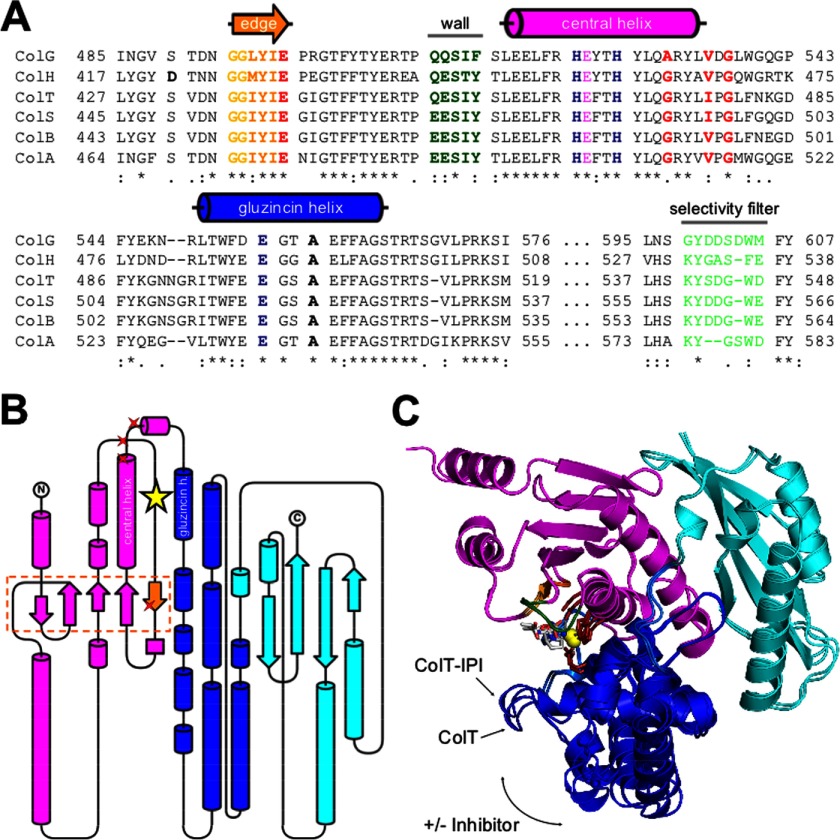
**Conservation and variation of functional and structural elements.** *A*, multiple sequence alignment of the catalytic center of six different clostridial collagenases: ColA from *C. perfringens* (UniProt entry code P43153); ColG from *C. histolyticum* (Q9X721); ColH from *C. histolyticum* (Q46085); ColB from *C. botulinum* (A7GDU8); ColS from *C. sporogenes* (Q84IM4); and ColT from *C. tetani* (Q899Y1). The zinc-binding residues shown in *dark blue* characterize the collagenases as gluzincins. The calcium-binding residues are *highlighted* in *red*, and the selectivity filter is shown in *light green*. The edge strand with the preceding double-Gly motif is shown in (*light*) *orange. B*, topology diagram of the peptidase domain. The upper and lower half-domains are shown in *magenta* and *blue*, respectively. The helper subdomain is shown in *cyan*. The catalytic zinc ion is depicted as a *yellow star*. Proteinaceous Ca^2+^-binding residues are indicated with *red stars*. The edge strand forming the non-primed substrate recognition sites is shown in *orange*. Artwork was done with TopDraw (47). *C*, *superimposition* of the peptidase domain of collagenase T in the presence and absence of the peptidic inhibitor isoamylphosphonyl-GPA. Inhibitor binding induces a globular domain movement of the upper (*magenta*) and lower (*blue*) half-domains that leads to a contraction of the active site cleft. The helper subdomain (*cyan*) acts as a platform that serves as a scaffold to the half-domain dynamics.

Based on a BLAST search, we found significant sequence similarities (*E* < 1 × *e*^−50^) for the ColG peptidase domain (Q9X721; Asp^398^–Gly^790^; 46 kDa) only toward clostridial and bacilli collagenases (38), consistent with previous analyses on the collagenase unit (39). The peptidase domains of ColH (Q46085; Pro^330^–Gly^721^; 46 kDa) and ColT (Q899Y1; Asp^340^–Lys^731^; 46 kDa) showed sequence identities of 56 and 55% and sequence similarities of 71 and 74%, respectively. Within these three peptidases, we found an even higher sequence conservation around the active site (>68% identity and >81% similarity) (*cf.* Fig. 2*A*), notwithstanding the strong variation of their enzymatic activities (10, 17, 39–41).

##### Protein Structure Quality

In order to understand the basis of the different enzymatic properties, we determined the crystal structures of the peptidase domains of ColH and ColT and the collagenase unit of ColG in the presence and absence of the peptidic inhibitor isoamylphosphonyl-GPA. The structures were determined in the resolution range from 1.7 to 2.2 Å with all residues being defined in electron density at excellent geometric and crystallographic parameters (Table 1). To verify the proposed calcium binding site in the PKD-like domain (22), the crystal structure of an N-terminally extended construct of the PKD-like domain of ColG (Asn^795^–Asn^880^) was determined in the presence of 10 mm calcium at 0.99 Å resolution.

**TABLE 1:** **X-ray data collection and model refinement statistics**

	Peptidase domain of ColH		Peptidase domain of ColT		Collagenase G		
	−Inhibitor	+Inhibitor	−Inhibitor	+Inhibitor	Collagenase unit	PKD-like domain Asn^795^–Asn^880^	PKD-like domain Ile^799^–Asn^880^*^a^*
**Data collection**							
Space group	P2_1_2_1_2	P2_1_2_1_2	P2_1_2_1_2_1_	P2_1_2_1_2_1_	P2_1_2_1_2_1_	P2_1_	C121
Cell dimensions							
*a* (Å)	79.1	79.9	76.9	74.4	55.3	19.8	68.2
*b* (Å)	108.2	106.8	102.1	102.1	108.7	70.9	59.3
c (Å)	51.3	51.4	104.8	102.5	181.0	23.4	55.3
α (degrees)	90.0	90.0	90.0	90.0	90.0	90.0	90
β (degrees)	90.0	90.0	90.0	90.0	90.0	95.2	125.6
γ (degrees)	90.0	90.0	90.0	90.0	90.0	90.0	90
Wavelength	0.9791	0.8856	0.8856	0.8856	0.8856	0.8856	0.98793
Resolution (Å)	46.33-2.01	40.00-1.77	39.85-1.69	45.70-2.05	69.50-2.19	35.44-0.99	40.51-1.18
*R*_merge_	0.135 (0.760)*^b^*	0.067 (0.404)	0.100 (0.804)	0.114 (0.587)	0.067 (0.604)	0.025 (0.064)	0.066 (0.299)
*I*/σ*I*	7.8 (2.0)	10.0 (2.0)	8.7 (2.0)	6.0 (2.0)	10.7 (2.0)	27.3 (12.1)	13.9 (3.2)
Completeness (%)	99.8 (99.6)	97.6 (86.2)	96.2 (94.5)	98.1 (98.5)	99.7 (99.6)	91.8 (77.1)	96.4 (78.4)
Redundancy	5.8 (5.2)	4.4 (3.1)	5.7 (5.6)	4.0 (4.0)	4.8 (4.7)	3.4 (3.1)	6.0 (2.9)
Wilson *B*-factor (Å^2^)	23.3	22.4	20.0	26.9	41.6	5.4	9.7

**Refinement**							
Resolution (Å)	46.33-2.01	40.00-1.77	39.85-1.69	45.70-2.05	69.50-2.19	35.44-0.99	40.51-1.18
No. of unique reflections	29,831	42,481	88,975	48,639	56,974	32,608	56,640
*R*_work_/ *R*_free_	20.03/24.95	15.02/20.01	17.23/22.10	21.76/26.79	19.37/24.20	12.23/14.78	14.73/18.00
No. of non-hydrogen atoms							
Protein	3106	3105	6350	6274	5362	674	1323
Ligand	2	2	4	22	41	1	-
Solvent	168	267	569	280	269	134	364
*B*-Factors							
Protein	30.98	29.22	22.55	36.69	63.01	8.33	13.93
Ligand	25.60	28.21	20.54	49.00	55.21	5.83	
Solvent	32.26	35.08	29.99	37.36	54.93	19.38	38.77
Root mean square deviations							
Bond lengths (Å)	0.006	0.008	0.012	0.005	0.008	0.013	0.018
Bond angles (degrees)	0.988	1.147	1.393	0.864	1.316	1.550	1.805

*^a^* Data were published previously (22).

*^b^* Values in parentheses are for the highest resolution shell.

##### Relations of the Peptidase Domain within the Protein Structure Universe

A global structural comparison of the peptidase domain with deposited protein structures revealed that structurally related proteins all belonged to the MA-Clan; we found the highest agreement with members of the M1 (alanine aminopeptidase/leukotriene A4 hydrolase) and M4 (thermolysin) family (4); in absolute terms, however, the structural agreement was rather low, with 175–210 aligned residues and *Z*-scores of 6.8–10.6, reflecting the low sequence identities of only 6–14%. As a reference, the three collagenase homologues agreed to a *Z*-score of 50 or higher.

Importantly, we could identify structural homologues in the protein database only for the N-terminal (upper and lower) catalytic subdomain of the collagenase peptidase domain; by contrast, we could not detect a significant structural relation for the single C-terminal helper subdomain, suggesting a collagenase-specific role for the latter. These findings are in agreement with our previous structural analysis whereby the peptidase domain of ColG was segmented into a catalytic and a helper subdomain (7). The peptidase domains of ColH and ColT showed an analogous segmentation into a catalytic subdomain and a helper subdomain (Figs. 1 and 2 (*B* and *C*)).

##### Partitioning of the Catalytic Subdomain

**FIGURE 3.**
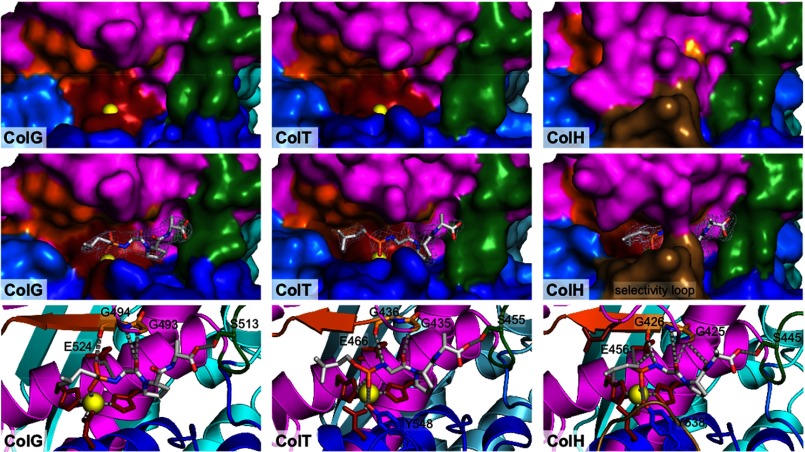
**Active site comparison of collagenase G, H, and T in the free and inhibitor-complexed state.** Characteristic recognition elements are indicated with identical *color coding* throughout and *labeled* for the free ColG. The catalytic zinc (shown in *yellow*) is accessible in free ColG and ColT but blocked in ColH by the Asp switch and is therefore not visible. The wall (*green*) serves as a molecular ruler, explaining the tricarboxypeptidase activity of clostridial collagenases. The inhibitor structures are *superimposed* with their experimental (2*F*_*o*_ − *F*_*c*_) electron density contoured at 1.0 σ over the mean. In a *close-up view* of the inhibitor-complexed state, the interactions of the inhibitor with its proteinaceous ligands next to the catalytic residues and the zinc ion are depicted.

The catalytic subdomain can be divided into an upper N-terminal and a lower C-terminal half-domain, both of which embrace the active site cleft. As described analogously for ColG (7), inhibitor binding induced a 2-Å contraction of these segments in ColT (Fig. 2*C*). This conformational breathing emphasizes the hingelike active site topology in clostridial collagenases. Although all peptidases adopted an open conformation in the unliganded apo-form and a more compact conformation in the complexed state, this breathing movement was most pronounced in ColG and least pronounced in ColH (Figs. 2*C* and 3).

The *helper subdomain* is composed of a central β-sheet that is flanked by four α-helices (Fig. 2, *B* and *C*). It strengthens the relative arrangement of the catalytic segments within the peptidase domain. The stabilizing interactions of the helper subdomain with the upper segment and, to a lower extent, with the lower catalytic segment are reflected by their contact areas of 1060 and 418 Å^2^ for ColG, 1046 and 347 Å^2^ for ColH, and 955 and 503 Å^2^ for ColT. The upper and lower segments form a 959-, 1115-, and 1359 Å^2^ contact area, respectively. Consequently, the contact area between the upper and lower catalytic subdomains is larger by 42% in ColT than in ColG. Similarly, the lower catalytic subdomain has more than 20% stabilizing interactions with the helper domain, together suggesting a considerably more rigid quaternary arrangement within the peptidase in ColT as compared with ColG and also ColH. This difference might point toward different substrate specificities in these enzymes; alternatively, the PKD domain(s) present in ColG/H might compensate for the additional quaternary stabilization found in ColT.

**FIGURE 4.**
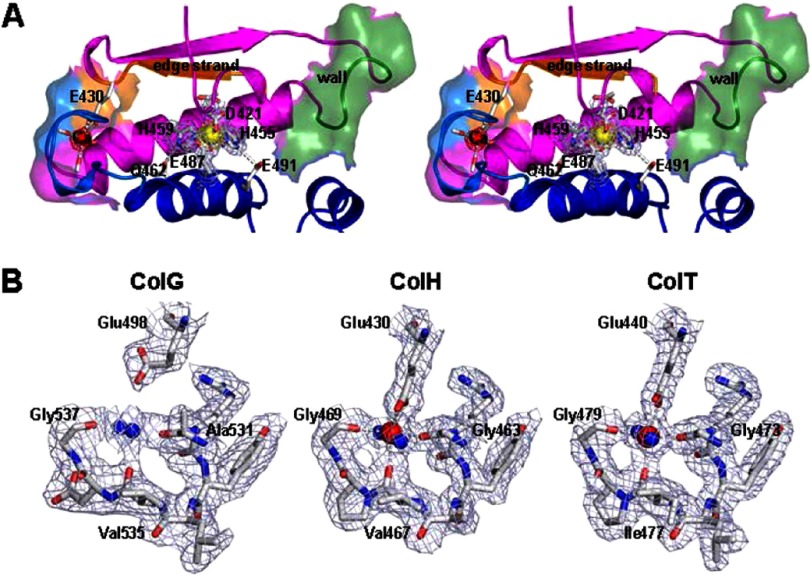
**Three-dimensional active site topology.** *A*, *wall-eyed stereo representation* of the active site of unliganded collagenase H with the Asp switch. Key active site elements, such as the central helix and gluzincin helix; the edge strand forming the non-primed substrate recognition; the Ca^2+^-binding site with carbonyl oxygen and Glu^430^ carboxylate ligands; the wall motif; and the two catalytic histidine-stabilizing glutamates are depicted. *B*, calcium binding site within the peptidase domain of ColG, -H, and -T. Due to >125 mm citrate in the crystallization buffer of the ColG crystals, the calcium site is occupied by a water molecule rather than by a Ca^2+^ ion. The conserved glutamate ligand (Glu^498^) is rotated away from the central solvent molecule. By contrast, in the Ca^2+^-loaded state, the glutamate contributes to an approximately octahedral coordination sphere around the cation. In all three proteases, the experimental (2*F*_*o*_ − *F*_*c*_) electron density is *overlaid*, contoured at 1.0 σ over the mean.

The *active site* is positioned between the upper and lower catalytic segments. The characteristic HE*XX*H consensus sequence is located on the central helix; the third proteinaceous zinc ligand is a glutamate located 28 residues (ColG and ColH) or 30 residues (ColT) downstream on the neighboring gluzincin helix (Fig. 2, *A* and *B*, and supplemental Table 1). The spatial arrangement of the zinc ligands is identical in all three peptidases, notwithstanding the difference in sequential separation. The two zinc-coordinating histidines are stabilized by a conserved glutamine and glutamate positioned one helical turn downstream of the HE*XX*H motif and the gluzincin ligand, respectively (Fig. 4).

Three amino acids downstream of the third zinc ligand, an alanine side chain (ColG, Ala^558^; ColH, Ala^490^; ColT, Ala^502^) serves as a hydrophobic basement ∼4.4 Å underneath the catalytic zinc. Its position and strict conservation in clostridial collagenases suggest a pivotal role of the hydrophobic basement for the active site core and function, as described in great detail for metzincins (42). The S1′ recognition site is formed by a conserved double glycine motif (Gly^493^-Gly^494^ in ColG); its two amide nitrogens orient and arrest the P1′ carbonyl oxygen in an oxyanion-type fashion, referred to as the secondary oxyanion pocket. Following the double Gly motif, Leu^495^–Glu^498^ form the non-primed substrate recognition strand. This so-called edge strand participates in a mixed five-stranded β-sheet and is composed of mostly hydrophobic residues; Glu^498^ anchors the edge strand by an ionic interaction with the calcium, as described below. The primed recognition site is delimited by a pronounced wall-like feature (Gln^511^–Phe^515^) positioned on the loop preceding the central helix. The active site topology thus allows the P5–P3′ substrate residues to bind in an extended conformation that is preferred for cleavage (43) (compare Fig. 3).

In addition to these shared active site determinants, we identified several discriminating features around the active site of the three homologous peptidases (Fig. 2*A* and supplemental Fig. 1). The most striking difference within the uncomplexed structures was identified in the peptidase domain of ColH, where Asp^421^ binds to the active site zinc (Fig. 4). Formally, this interaction mode is reminiscent of the aspartate switch in proastacin (44). This interaction was not observed in ColG and ColT, which have a serine at the position of the Asp^421^ in ColH (Fig. 2*A*). This ionic interaction completely rearranged the segment Asn^414^–Asn^424^ but left the substrate recognition elements, such as the double-glycine motif (positions 425 and 426) and the edge strand (Met^427^–Glu^430^), unaffected. Upon inhibitor binding, Asp^421^ got displaced and adopted a conformation similar to that in ColG and ColT (supplemental Fig. 1).

Additionally, we found that the segment Lys^530^–Glu^536^ in ColH folded into a prominent loop structure that selects access to the active site (Fig. 3 and supplemental Fig. 1). The conformation of this selectivity loop is virtually identical in the complexed and uncomplexed ColH structure. Thereby, Tyr^531^ and Phe^537^ can interact with and stabilize other active site residues. This interaction results in a reaction tube-like compartmentalization of the active site. The combination of the aspartate switch and the selectivity loop renders the active site nearly completely inaccessible in the uncomplexed structure of ColH (Fig. 3). This selectivity loop was unique to ColH and may be related to the charged character of the corresponding segment in ColT and ColG, which possess one and three aspartate residues.

##### Peptidase Calcium Binding Site

Near the S5 site defined by the edge strand (Pro^499^) and ∼15 Å apart from the catalytic zinc, we could identify a structurally conserved calcium binding site (Fig. 4*B*). The calcium ion was octahedrally coordinated by two water molecules, three backbone oxygens (ColG, Ala^531^, Val^535^, and Gly^537^), and a conserved glutamate side chain (ColG, Glu^498^) (*cf.* Figs. 2*A* and 4). The latter partially coordinated the calcium in a bidentate manner, thus providing a seventh ligand to the calcium, albeit at a greater distance. Further details are provided in supplemental Table 2. In the ColG crystal structures, this site remained unloaded because of the presence of at least 125 mm citrate in the crystallization buffer. As evidenced by the comparison of Ca^2+^-unloaded (ColG) and -loaded (ColH/T) crystal structures, the liganding glutamate side chain undergoes a marked rearrangement upon Ca^2+^ binding that allows Ca^2+^ coordination. Due to its location between the two catalytic helices, calcium binding braces the upper and lower half-domains of the peptidase and stabilizes thereby the zinc coordination.

##### Calcium Binding Site of the PKD-like Domain

**FIGURE 5.**
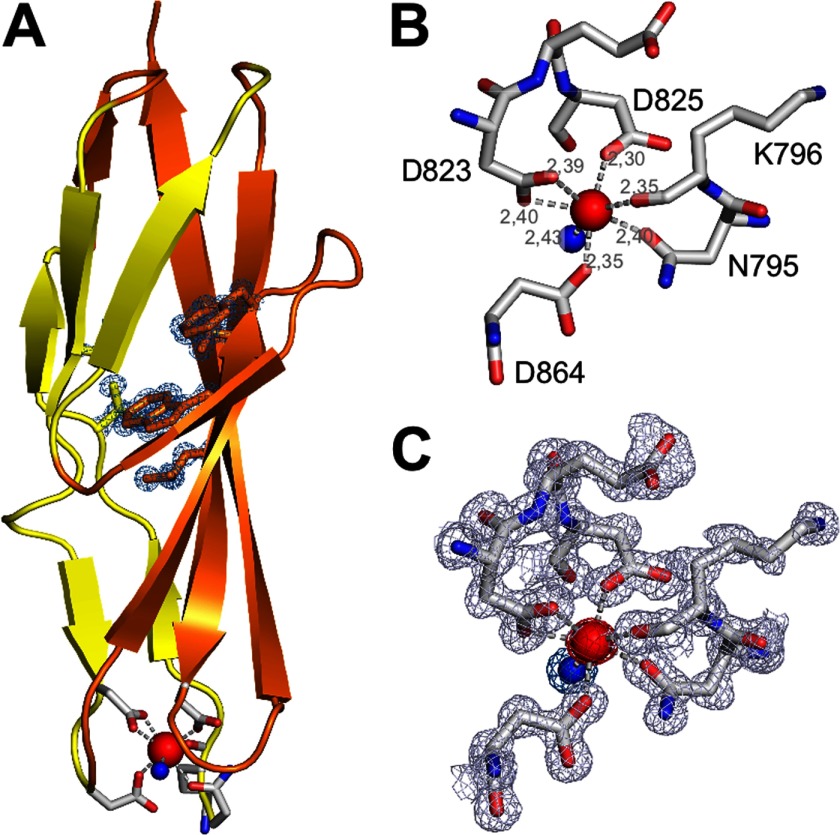
**Calcium binding site within the PKD-like domain.** *A*, overall structure of the PKD-like domain of ColG. The N-terminally located Ca^2+^-binding site is shown in a *ball-and-stick representation*. The side chains of the hydrophobic core (Val^802^, Phe^816^, Phe^835^, Val^858^, and Leu^860^) are represented in *sticks* and *overlaid* by the experimental (2*F*_*o*_ − *F*_*c*_) omit map, contoured at the 1 σ over the mean. *B*, *zoom-in view* to the calcium site, with seven ligands forming a pentagonal-bipyramidal coordination sphere. Asp^823^ coordinates the calcium within the pentagonal plane in a bidentate manner. The proteinaceous ligands are *labeled*, and metal distances are indicated. *C*, *zoom-in view* of the calcium site with the experimental (2*F*_*o*_ − *F*_*c*_) electron density *overlaid*, indicating the excellent quality of the density.

Although the previously reported ColG crystals contained the PKD-like domain in addition to the collagenase unit, the PKD-like domain remained largely disordered (7). This disorder is presumably related to the presence of citrate in the crystallization buffer, which titrates away Ca^2+^ ions. Because the crystal packing did not tolerate rebuffering to introduce calcium, we produced and crystallized an isolated PKD-like domain spanning residues Asn^795^–Asn^880^ to investigate its calcium-binding properties. In this orthorhombic crystal form, we identified six proteinaceous ligands, namely Asn^795^, Lys^796^, Asp^825^, Asp^864^, and both of the carboxylate oxygens of Asp^823^, which together with a seventh water ligand coordinate the calcium ion in a pentagonal-bipyramidal geometry (Fig. 5). Detailed coordination geometries are summarized in supplemental Table 2. A previously described monoclinic crystal form of a PKD variant lacking four amino acids (Ile^799^–Asn^880^) did not bind calcium (22). It is noteworthy that the overall structure and the behavior of the protein during expression, purification, and biophysical characterization remained identical; this indicated that calcium binding induces only local rearrangements at the N terminus of the PKD-like domain.

In addition, an N-terminally further extended PKD construct (Ile^792^–Asn^880^) was crystallized in the same conditions as the Asn^795^–Asn^880^, but the additional residues (Ile^792^, Ser^793^, and Asn^794^) could not be traced in the electron density, indicating their flexibility even in the presence of calcium (data not shown). Notably, in both PKD variants (Ile^792^–Asn^880^ and Asn^795^–Asn^880^), the calcium could not be extracted by incubating the respective crystals for up to 4 h with 100 mm EDTA.

##### Enzymatic Characterization

**FIGURE 6.**
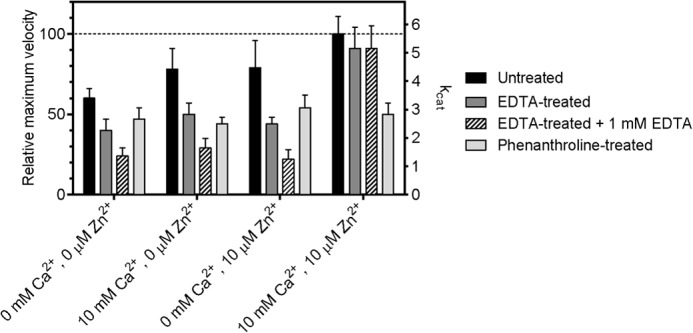
**Metal dependence of the enzymatic activity of the ColT peptidase domain.** Shown are the maximum velocity (*V*_max_) and turnover number (*k*_cat_) of collagenase T for the substrate FALGPA and a comparison of untreated ColT with EDTA- and 1,10-phenanthroline-pretreated ColT. Pretreated protein samples were incubated overnight with 10 mm EDTA or 10 mm 1,10-phenanthroline for ion extraction. All protein samples were rebuffered using NAP-desalting columns into the reaction buffer containing the indicated ion concentrations. In the case of EDTA-pretreated protein, measurements were performed in reaction buffer containing 0 and 1 mm EDTA, respectively. The *k*_cat_ (s^−1^) and the relative *V*_max_ (%) values were determined by nonlinear regression. Mean and S.D. values are indicated.

We selected the peptidase domain of ColT for enzymatic characterization in order to eliminate effects presumably caused by the aspartate switch (*e.g.* large variations of single measurements) in ColH, whereas ColG is the collagenase homologue with the lowest amidolytic activity (5). Even with ColT, we observed significant variations (*cf. error bars* in Fig. 6 and supplemental Fig. 2). After an extended series of kinetic experiments, we came to conclude that these variations reflect an important intrinsic property of this enzyme rather than an experimental error, as will be explained below.

The data showed that ColT could not be fully inactivated by metal-chelating reagents, such as EDTA or phenanthroline. Preincubation of ColT with phenanthroline irreversibly reduced the activity, albeit not completely (Fig. 6). Phenanthroline is thought to chelate and extract the catalytic zinc, which appears to be effective only in half of the proteases; once extracted, the active site cannot be reconstituted by adding metals (zinc, calcium, or a combination of both metals). Contrasting with the phenanthroline inhibition, EDTA reduced *V*_max_ reversibly (Fig. 6). The effect is strongest when considering ColT, which was inhibited with EDTA in the reaction buffer, where *V*_max_ was reduced to <25%. Importantly, each individual metal had only marginal effects, ∼20% enhancement for Ca^2+^ and ∼0% enhancement for Zn^2+^ (with errors overruling these effects). Only the simultaneous presence of both metals significantly increased *V*_max_ by ∼300% (Fig. 6).

These findings are puzzling at first sight but can be reconciled when assuming two different zinc-depleted peptidase conformations. The interplay of these conformational substates is described under “Discussion.”

It is noteworthy that the gel filtration-purified but otherwise untreated sample showed only 50% of the maximum velocity, indicating a partial loss of one or both metals upon size exclusion chromatography. Again, 10 mm Ca^2+^ together with 10 μm Zn^2+^ could restore 100% activity, but neither of the metals alone had a significant effect.

Furthermore, metal extraction (via EDTA or phenanthroline) or addition had only small or insignificant effects on the *K*_*m*_ (supplemental Fig. 2*A*). This suggests that metal extraction affects the active site geometry only locally, leaving major substrate recognition elements like the edge strand undisturbed (Fig. 4*A* and supplemental Fig. 1).

## DISCUSSION

### 

#### 

##### Domain Character of the Peptidase

The comparison of the isolated peptidase domains of ColH and ColT with the crystal structure of ColG containing the full collagenase unit plus the PKD-like domain reveals that the peptidase structure is unaffected by the contiguous domains (Fig. 1). This observation suggests that the peptidase is a true domain; this conclusion is corroborated by the facts that the peptidase (i) adopts a compact three-dimensional structure, (ii) is capable of independently folding, and (iii) can function as a peptidase independently of the rest of the protein.

##### Dual Function for the Helper Subdomain

The peptidase domain could be further segmented into the upper and lower half-domains, which adopt a thermolysin fold, and a C-terminal helper subdomain. The latter appears to stabilize the relative arrangement of the thermolysin-like half-domains and thus controls the breathing motion concomitant with substrate binding (Figs. 2*C* and 3 and supplemental Fig. 1). This interpretation is in line with the reported inactivity of a collagenase construct lacking the helper subdomain (5). The core of the helper subdomain resembles the structure of sheet B in the PKD-like domain (22). This structural relation suggests an additional function of the helper subdomain, whereby the helper subdomain acts as a platform to direct the three-dimensional arrangement of the downstream recruitment domains via a sheet extension mechanism. Such a mechanism was suggested to control the relative positioning within the collagen recruitment domains (7, 22).

##### Structural Relation within ColG, -H, and -T Reflects Their Substrate Specificities and Preferences

Notably, the peptidase domain of ColG from *C. histolyticum* showed a higher structural similarity to ColT from *C. tetani* than to ColH from *C. histolyticum*. This structural relation is in particular evidenced by the extent of peptidase half-domain motions accompanying inhibitor binding and may, on a functional level, reflect the substrate specificities of the three homologous collagenases. The interaction areas between the inhibitor isoamylphosphonyl-GPA with the individual enzymes is largest in ColH (256 Å^2^) > ColT (248 Å^2^) > ColG (220 Å^2^), as calculated using the PISA server (34). This relation explains the need of ColG for N-terminally extended peptides to obtain full peptidolytic activity (12).

##### Calcium and Zinc Are Both Required to Reconstitute the Activity of the Metal-depleted Peptidase

So far, only calcium binding sites within the CBD and the PKD-like domain have been identified (14, 22). Although the calcium binding within the recruitment domains could explain their relevance for protein stability and interdomain arrangement, it could not account for its critical role in enzymatic activity, because the collagenase unit of ColG is capable of collagen degradation without its additional C-terminal domains (7). By contrast, the now identified calcium binding site within the peptidase domain is in 15-Å proximity to the active site, (Fig. 4*A* and supplemental Fig. 1). Upon calcium binding, the upper and lower segments of the peptidase domain are clamped together, stabilizing the conformation of the ligands around the catalytic zinc ion. Conversely, the catalytic zinc tethers the peptidase half-domains and thus preforms the calcium binding site within the peptidase domain. Within this structural framework it is understandable how zinc and calcium can cooperate in activity enhancement, as observed in Fig. 6. Nonetheless, some of the observed effects may appear counterintuitive at first sight, including the role of zinc; the addition of zinc with or without calcium could not restore the activity of phenanthroline-treated ColT, suggesting that zinc could not be reconstituted. By contrast, zinc in addition to calcium is crucial to recover the activity of EDTA-treated ColT. These seemingly contradictory effects can be reconciled when proposing two distinct zinc-depleted conformations, one being irreversibly collapsed (phenanthroline-treated) and the other one having zinc replaced by water with the zinc-chelating residues still in place, as observed in ColG (7). In the latter case, zinc was gently removed and replaced by a water molecule by citrate in the surrounding crystallization solution, with the geometry of the zinc-coordinating residues remaining in place. In this situation, the catalytic zinc can be reloaded, albeit only in the presence of calcium. By contrast, the phenanthroline-extracted zinc cannot be reconstituted. We propose that phenanthroline binds directly to the zinc in the active site. By doing so, it will disturb the zinc coordination sphere, which will help to extract the phenanthroline-bound zinc from the active site. Moreover, we propose that by the phenanthroline interaction, some of the zinc-coordinating residues will be irreversibly reoriented (*e.g.* the HE*XX*H motif, Glu^487^, or second layer residues Gln^462^ and Glu^491^; *cf.* Fig. 4).

##### The Aspartate Switch in ColH Stabilizes and Protects the Catalytic Zinc in the Absence of Substrates

Another surprising observation was that ColT is only partially inhibited when preincubated with an excess of phenanthroline or EDTA. Why would a complete inactivation require EDTA to be present in the reaction buffer? Again, we can only explain these phenomena when invoking different conformational substates. We could crystallize ColH in the absence of substrate analogues in a closed conformation, involving the aspartate switch Asp^421^ (ColT; supplemental Fig. 1 and Fig. 4). Here, we found that Asp^421^ coordinates the catalytic zinc in addition to the proteinaceous ligands (His, His, and Glu) present in all gluzincins, including ColG and ColT. Importantly, this closed conformation is loaded with both zinc and calcium but renders them inaccessible to the solvent. In particular, this conformation cannot be modified by phenanthroline, and we conclude that ∼50% of the ColT population will adopt a closed conformation as crystallized and seen in ColH. It is further sensible to assume that the compressed conformation will also bind calcium more tightly, explaining why the same population should retain calcium even when occupied with EDTA.

The situation becomes more dynamic in the presence of substrates that will induce an open and active yet metal-vulnerable state. In this state, spontaneous metal bleeding will take place that is further driven by the presence of EDTA, explaining the steady activity reduction in the presence of EDTA during substrate turnover (Fig. 6 and supplemental Fig. 2*B*). The overlay of different active site conformations with different metal contents can also explain the relatively large measurement variations.

##### Aspartate Switch in Related Zinc Proteases

The here described aspartate switch is reminiscent of a similar zinc coordination in proastacin (44). The comparison allows for some interesting conclusions as to the possible relevance of the aspartate switch. In particular, the comparison suggests an autoinhibitory mechanism that confers latency by blocking substrate access to the active site.

Although the formal parallelism with proastacin may support the autoinhibition hypothesis, the fact that ColH is a highly active peptidase supports the zinc stabilization hypothesis analogous to the tyrosine switch in active astacin (45). This notion is further supported by the contracted quaternary arrangement of the peptidase half-domains in ColH that is induced by binding of Asp^421^ to the active site zinc (Fig. 1).

A further interesting analogy can be found when comparing open and closed states in ColH and ADAMTS4 (46). In ColH, both the active (open) and the closed states were crystallized in the presence of calcium, which is positioned at the non-primed site. In ADAMTS4, the occupancy of the calcium near the active site correlates with the open conformation (*i.e.* calcium binding and the closed form appear incompatible in ADAMTS4) (46). This conformational reorganization is driven by the presence or absence of a substrate-like ligand binding to the active site, resembling the situation in ColH (and presumably also ColT, as suggested by enzymatic data), where the loop opening also appears to be induced by the substrate.

The active site of ColH remains rather restricted even in the inhibitor-complex structure with the Asp^421^ displaced. This restriction mostly arises from a substrate selectivity loop that is characteristic of ColH and consistently explains the low collagenolytic activity, because triple helical substrates cannot easily access the restricted active site of ColH.

##### Preferences for Triple Helical Collagen Cleavage

On the one hand, the peptidase domains described here are insufficient to explain the collagenolytic properties of the full-length proteins, simply because they are unable to cleave collagen (7). On the other hand, we believe that the comparison of well characterized peptidolytic and collagenolytic activities of ColG and ColH point to structural features that are encoded already in the peptidase domain and discriminate their collagenolytic potency. Access to the active site is starkly restricted in ColH by a prominent “selectivity loop” as compared with ColG (Fig. 3 and supplemental Fig. 1). This difference in accessibility correlates with the preference of single chain peptidic substrates over triple helical collagen in ColH *versus* ColG (10). However, an activator domain is required in addition to the peptidase domain for collagen cleavage. It is important to consider the extensive hydration shell around triple helical collagen, the structure of which will be stabilized by the water molecules. We further proposed that the activator and peptidase domains embrace the collagen substrate, thereby stripping many of the collagen-associated water molecules (7). This will lead to a destabilization of collagen and may in consequence lead to presenting vulnerable collagen sites to the peptidase active site.

##### Conclusions

The comparison of three clostridial collagenase structures allows us to rationalize distinct substrate specificities and to engineer desired peptidolytic activities. The disclosure of unexpected regulatory principles can be employed to tune the enzymatic activity for different applications. Among them, the most important adjustments can be gained by controlling the aspartate switch and the metal-loaded state. Further, the substrate selectivity filter should allow the modification of both peptidolytic and collagenolytic activities. Finally, the structures will also support the rational design of clostridial collagenase inhibitors.

## Supplementary Material

Supplemental Data

## Footnotes

4 The abbreviations used are:

ColG, -A, -B, -H, -S, and -T: collagenase G, A, B, H, S, and T, respectively
PKD: polycystic kidney disease
CBD: collagen binding domain
FALGPA: *N*-[3-(2-furylacryloyl)]-l-leucyl-glycyl-l-prolyl-l-alanine
Tricine: *N*-[2-hydroxy-1,1-bis(hydroxymethyl)ethyl]glycine.
