# Supplementary material for: Structural Basis for Activity Regulation and Substrate Preference of Clostridial Collagenases G, H, and T
Source: J Biol Chem. 2013 May 23;288(28):20184–94. doi: 10.1074/jbc.M112.448548 (PMC3711286; doi:10.1074/jbc.M112.448548)
Supplement: Supplemental Data [file supp_288_28_20184__index.html]

Structural basis for activity regulation and substrate preference of clostridial collagenases G, H, and T — Structural Basis for Activity Regulation and Substrate Preference of Clostridial Collagenases G, H, and T — Crystal Structures of Clostridial Collagenases — Supplemental Data 

# Structural Basis for Activity Regulation and Substrate Preference of Clostridial Collagenases G, H, and T

## Supplemental Data

**Files in this Data Supplement:**

- Supplemental Table 1 (.pdf, 15 KB) - Functionally relevant elements. Distinct structural features of ColG, ColH and ColT and their amino acid positions are depicted (numbering according to the respective UniProt entry: ColG: Q9X721, ColH: Q46085, ColT: Q899Y1).
- Supplemental Table 2 (.pdf, 72 KB) - Metal binding sites of clostridial collagenases.
- Supplemental Figure 1 (.jpg, 231 KB) - Active site comparison of clostridial collagenases. Active site representation of ColG, H, and T in the unliganded and the inhibitor complexed state. Orientation is identical with Figure 3. The upper (magenta) and lower (blue) catalytic half-domains and the helper subdomain are shown in ribbon respresentation. The catalytic zinc ion (yellow) and the coordinating water molecule (blue) are shown as spheres, and the catalytic residues are shown in ball-and-stick representation. The edge strand with the preceding double-Gly motif is indicated in (light)orange. Note in particular the Ca2+ site loaded in ColT and ColH which is hidden in the solid surface representation of Figure 3.
- Supplemental Figure 2 (.jpg, 267 KB) - Enzymatic activity of the ColT peptidase domain in absence and presence of calcium and zinc and comparison of untreated ColT with EDTA- and 1,10-phenanthroline-pretreated ColT. A) Michaelis-Menten constants of the ColT peptidase domain for FALGPA. Experiments were performed as described in Figure 6. KM values were determined by nonlinear regression. Mean and standard deviation are indicated. B) Enzymatic activity of the ColT peptidase domain at 0.98 mM FALGPA. Protein preparation and measurements were performed as described. In case of EDTA-pretreated protein, additional measurements containing 10 mM EDTA in the reaction buffer were performed. Mean and standard deviation are indicated.
