# Supplementary material for: Structural Basis for Activity Regulation and Substrate Preference of Clostridial Collagenases G, H, and T
Source: J Biol Chem. 2013 May 23;288(28):20184–94. doi: 10.1074/jbc.M112.448548 (PMC3711286; doi:10.1074/jbc.M112.448548)
Supplement: Supplemental Data [file supp_M112.448548_jbc.M112.448548-3.pdf]

Supplemental Table 1. Sequence features of clostridial collagenases

	Collagenase G			Collagenase H			Collagenase T		
**Asp-switch**	487	GVSTDN	492	419	GY**D**TNN	424	429	GYSVDN	434
**Double-Gly motif Edge strand**	493	GG LYIE	498	425	GG MYIE	430	435	GG IYIE	440
**Wall motif**	511	QQSIF	515	443	QESTY	447	453	QESIY	457
**Central helix**	517	LEEL...ARYL	534	449	LEEL...GRYA	466	459	LEEL...GRYL	476
**Ca^{2+}-binding**	498	**E**...**A**RYL**V**D**G**	537	430	**E**...**G**RYA**V**P**G**	469	440	**E**...**G**RYL**I**P**G**	479
**Zinc-binding**	523	**H**EYT**H**...**E**	555	455	**H**EYT**H**...**E**	487	465	**H**EFT**H**...**E**	499
**Gluzincin helix**	550	LTWF...FFAG	563	482	LTWY...LFAG	495	494	ITWF...FFAG	507
**Selectivity loop**	598	GYDDSDWM*	605	530	KYGASFE	536	540	KYSDGWD*	546

* Residues corresponding, but not found to form a selectivity loop.
