# Supplementary material for: Structural Basis for Activity Regulation and Substrate Preference of Clostridial Collagenases G, H, and T
Source: J Biol Chem. 2013 May 23;288(28):20184–94. doi: 10.1074/jbc.M112.448548 (PMC3711286; doi:10.1074/jbc.M112.448548)
Supplement: Supplemental Data [file supp_M112.448548_jbc.M112.448548-4.pdf]

Supplemental Table 2 Metal binding sites of clostridial collagenases

A) Zinc binding site of clostridial collagenases

**ColH peptidase domain**			
		**- Inhibitor**	**+ Inhibitor**
**Resolution (Å)**		2.01	1.77
**Zn^{2+}**	**Occ**	1.0	1.0
	**B-fac**	26.1	20.8
**His455**	**Dist**	2.03	2.06
	**B-fac**	25.7	20.3
**His459**	**Dist**	2.03	2.03
	**B-fac**	26.8	22.0
**Glu487**	**Dist**	1.99	2.00
	**B-fac**	24.2	21.2
**Asp421**	**Dist**	2.01	-
	**B-fac**	34.5	-
**IPI**	**Dist**	-	1.99
	**B-fac**	-	22.9

**ColT peptidase domain**					
		**- Inhibitor[A]**	**- Inhibitor[B]**	**+ Inhibitor[A]**	**+ Inhibitor[B]**
**Resolution (Å)**		1.69	1.69	2.05	2.05
**Zn^{2+}**	**Occ**	1.0	1.0	1.0	1.0
	**B-fac**	33.8	20.5	34.0	38.1
**His465**	**Dist**	2.04	2.03	2.03	2.04
	**B-fac**	26.3	18.1	32.4	34.0
**His469**	**Dist**	2.03	2.03	2.03	2.03
	**B-fac**	17.0	16.7	29.5	33.7
**Glu499**	**Dist**	2.00	1.99	1.99	1.99
	**B-fac**	23.3	21.4	28.2	34.2
**H_2O**	**Dist**	2.03	2.02		
	**B-fac**	37.3	24.6		
**IPI**	**Dist**	-	-	2.03 | 2.24	2.03
	**B-fac**	-	-	42.5 | 34.5	44.7

**ColG collagenase unit**			
		**- Inhibitor**	**+ Inhibitor***
**Resolution (Å)**		2.19	3.25
**Zn^{2+}**	**Occ**	1.0	1.0
	**B-fac**	52.7	55.58
**His523**	**Dist**	2.09	2.08
	**B-fac**	53.8	63.03
**His527**	**Dist**	2.11	2.09
	**B-fac**	43.1	60.4
**Glu555**	**Dist**	2.00	2.09
	**B-fac**	57.1	85.0
**H_2O**	**Dist**	2.08	2.07
	**B-fac**	40.7	58.3
**IPI**	**Dist**	-	2.08
	**B-fac**	-	72.1

[A,B] Monomer A and B of the asymmetric unit.

* Based on data published previously (7).

Supplemental Table 2 Metal binding sites of clostridial collagenases

B) Calcium binding sites of clostridial collagenases

ColH peptidase domain			
		- Inhibitor	+ Inhibitor
Resolution (Å)		2.01	1.77
Ca^{2+}	Occ	1.0	1.0
	B-fac	25.1	19.1
Glu430	Dist	2.21 | 2.43	2.34
	B-fac	27.2 | 26.4	20.8
Gly463	Dist	2.36	2.32
	B-fac	23.9	19.8
Val467	Dist	2.37	2.31
	B-fac	25.1	20.9
Gly469	Dist	2.35	2.31
	B-fac	24.9	22.6
H_2O_1	Dist	2.18	2.36
	B-fac	21.7	20.5
H_2O_2	Dist	2.67	2.41
	B-fac	24.4	18.2

ColT peptidase domain					
		- Inhibitor[A]	- Inhibitor[B]	+ Inhibitor[A]	+ Inhibitor[B]
Resolution (Å)		1.69	1.69	2.05	2.05
Ca^{2+}	Occ	1.0	1.0	1.0	1.0
	B-fac	13.5	14.4	25.2	30.0
Glu440	Dist	2.32	2.31	2.31	2.33
	B-fac	18.9	15.7	24.7	33.3
Gly473	Dist	2.35	2.35	2.32	2.32
	B-fac	13.7	15.2	22.5	26.1
Ile477	Dist	2.35	2.34	2.32	2.32
	B-fac	14.7	14.3	22.9	29.9
Gly479	Dist	2.34	2.35	2.32	2.32
	B-fac	17.7	15.9	24.8	32.3
H_2O_1	Dist	2.36	2.39	2.35	2.34
	B-fac	16.5	13.4	27.1	28.7
H_2O_2	Dist	2.40	2.40	2.35	2.39
	B-fac	15.0	13.8	22.7	28.5

ColG PKD-like domain		
Resolution (Å)		0.99
Ca^{2+}	Occ	1.0
	B-fac	5.8
Asn795	Dist	2.43
	B-fac	7.5
Lys796	Dist	2.36
	B-fac	5.8
Asp823	Dist	2.42 | 2.43
	B-fac	8.2 | 7.5
Asp825	Dist	2.29
	B-fac	7.3
Asp864	Dist	2.35
	B-fac	5.4
H_2O	Dist	2.43
	B-fac	8.5

[A,B] Monomer A and B of the asymmetric unit.
