# Supplementary figures and images for: Structural Basis for Activity Regulation and Substrate Preference of Clostridial Collagenases G, H, and T
Source: J Biol Chem. 2013 May 23;288(28):20184–94. doi: 10.1074/jbc.M112.448548 (PMC3711286; doi:10.1074/jbc.M112.448548)

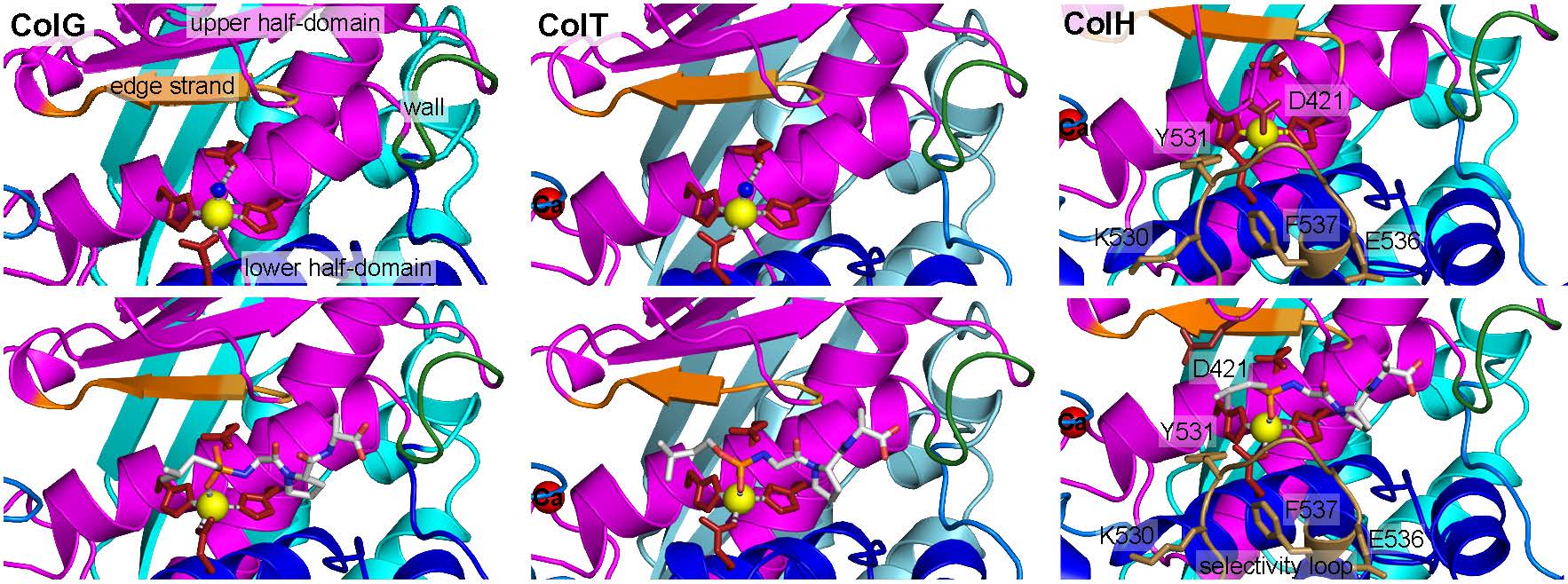

Supplement: Supplemental Data [file supp_M112.448548_jbc.M112.448548-1.jpg]

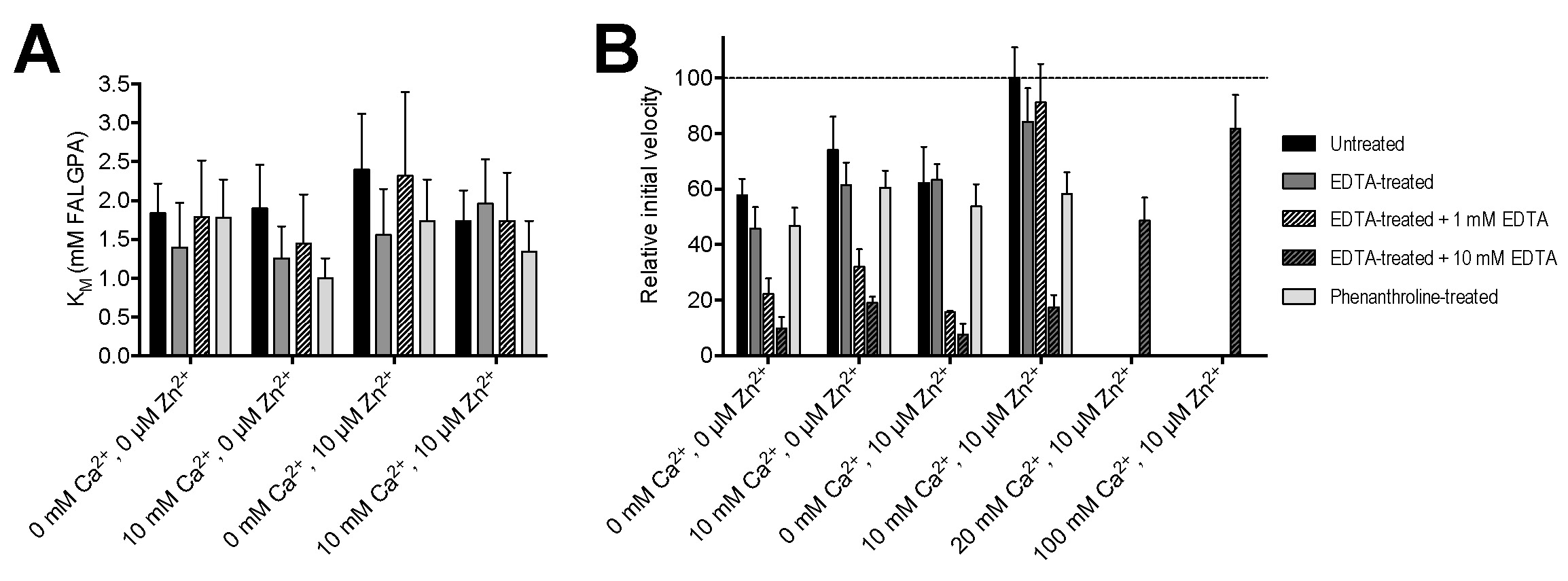

Supplement: Supplemental Data [file supp_M112.448548_jbc.M112.448548-2.jpg]
